# Physician satisfaction with a multi-platform digital scheduling system

**DOI:** 10.1371/journal.pone.0174127

**Published:** 2017-03-22

**Authors:** Rodrigo Octávio Deliberato, Leonardo Lima Rocha, Alex Heitor Lima, Caroline Reis Maia Santiago, Jose Cláudio Cyrineu Terra, Alon Dagan, Leo Anthony Celi

**Affiliations:** 1 Critical Care Department, Hospital Israelita Albert Einstein, São Paulo, Brazil; 2 Innovation Department, Hospital Israelita Albert Einstein, São Paulo, Brazil; 3 Laboratory of Computational Physiology, Harvard-MIT Health Sciences & Technology, MIT, Cambridge, Massachusetts, United States of America; 4 Department of Emergency Medicine. Beth Israel Deaconess Medical Center, Boston, Massachusetts, United States of America; 5 Department of Medicine. Beth Israel Deaconess Medical Center, Boston, Massachusetts, United States of America; Northwestern University, UNITED STATES

## Abstract

**Objective:**

Physician shift schedules are regularly created manually, using paper or a shared online spreadsheet. Mistakes are not unusual, leading to last minute scrambles to cover a shift. We developed a web-based shift scheduling system and a mobile application tool to facilitate both the monthly scheduling and shift exchanges between physicians. The primary objective was to compare physician satisfaction before and after the mobile application implementation.

**Methods:**

Over a 9-month period, three surveys, using the 4-point Likert type scale were performed to assess the physician satisfaction. The first survey was conducted three months prior mobile application release, a second survey three months after implementation and the last survey six months after.

**Results:**

51 (77%) of the physicians answered the baseline survey. Of those, 32 (63%) were males with a mean age of 37.8 ± 5.5 years. Prior to the mobile application implementation, 36 (70%) of the responders were using more than one method to carry out shift exchanges and only 20 (40%) were using the official department report sheet to document shift exchanges. The second and third survey were answered by 48 (73%) physicians. Forty-eight (98%) of them found the mobile application easy or very easy to install and 47 (96%) did not want to go back to the previous method. Regarding physician satisfaction, at baseline 37% of the physicians were unsatisfied or very unsatisfied with shift scheduling. After the mobile application was implementation, only 4% reported being unsatisfied (OR = 0.11, p < 0.001). The satisfaction level improved from 63% to 96% between the first and the last survey. Satisfaction levels significantly increased between the three time points (OR = 13.33, p < 0.001).

**Conclusion:**

Our web and mobile phone-based scheduling system resulted in better physician satisfaction.

## Introduction

One of the major challenges facing medical centers around the world is the ever more complicated task of physician scheduling [1]. Every day physicians are dealing with multiple schedules: their personal appointments, their research responsibilities, and their clinical shifts. Often these professionals work in multiple clinical settings over different periods of time. Still, it is very common for shift schedules to be under the jurisdiction of a person, often using some calendar on paper or as a “gatekeeper” of an online spreadsheet. The process of constructing a schedule or accepting a shift exchange, is complex, time consuming and requires the physicians to use multiple communication tools including emails, phone calls and messages.

The scheduling systems in current use are prone to mistakes that lead to scheduling conflicts, last minute scramble to cover a shift and physician dissatisfaction. Prior analysis of physician scheduling systems has concluded that an important area for improvement is the use of a computerized system [1]. The hope is that technology would reduce time wasted and prevent scheduling errors [2–4].

The scarce literature that does exist on the topic focuses on mathematical modeling approaches to constraint satisfaction problems and typically limited to patient throughput in the emergency department or the operating rooms. None of these previous studies have evaluated physician scheduling systems and measured user experience and satisfaction [5–7].

Smartphones and mobile applications have recently become ubiquitous in healthcare. According to the United Nations there are more than 3.6 billion mobile-broadband subscriptions worldwide in 2016 [8]. In the US and UK, around 90% of the physicians own a smartphone. More than 50% of physicians with a smartphone have downloaded medical apps, and of these, 70% use them regularly [9–12].

We developed a novel multi-platform physician scheduling system to replace the traditional centralized calendar or spreadsheet model. The project was chosen to be one of the pilot projects of the newly formed Innovation Department at the Hospital Israelita Albert Einstein (HIAE), a 657 bed quaternary private hospital, with more than 31,000 hospital admissions per year located in São Paulo, Brazil. This department is founded on collaboration between physicians and technology experts, both software and hardware, with physician involvement throughout the life cycle of every project. Within the framework of this cross-disciplinary department, we developed a new digital solution which streamlines the physician scheduling process, that allows users to access and switch shifts on a mobile application.

In this study, we describe the mobile application component of the scheduling system and measure physician satisfaction at different time points during implementation.

## Materials and methods

### Setting

This study was performed at the critical care department of HIAE between November 2015 and August 2016. This department includes two mixed (medical, surgical, coronary and cardiothoracic) ICUs and four intermediate care units with a total of 130 critical care beds and 10,200 admissions per year. The department has 66 board-certified physicians. All of them were invited to participate in the study. There were no exclusion criteria. Participation in the study was voluntary and there were no financial incentives. The study protocol was approved by the Institutional Review Board of HIAE and all participants filled a consent form prior to their participation.

### The previous working shift schedule model

Previous to the study, the working shift schedule was created and released monthly by the administrative staff as an online spreadsheet, taking into consideration department guidelines and physician requests. Each daily schedule included 10 physicians on day shifts and 6 physicians on night shifts. Each physician was scheduled for two to three 12-hour shifts (day and/or night) per week. Any changes or requests after the schedule was published would have to be negotiated on a physician-to-physician basis and then hand written on a spreadsheet located in the department office.

### The new physician scheduling system and the mobile application

The scheduling software and the mobile application were developed by the Innovation Department at HIAE with close collaboration between the developers and physicians, using the Lean Startup methodology [13]. The solution consists of two different tools: a web interface for the administrative staff (https://escala.inovaeinstein.com.br/painel/) to construct the schedule based on the departmental guidelines, and a mobile application for physicians to access the most updated schedule and request shift exchanges.

The web interface was developed using PHP, JavaScript, CSS and HTML. The server is hosted by an Elastic Beanstalk at Amazon Web Services (AWS). Data is entered into a MySQL also relational database hosted at AWS.

**Fig 1:**
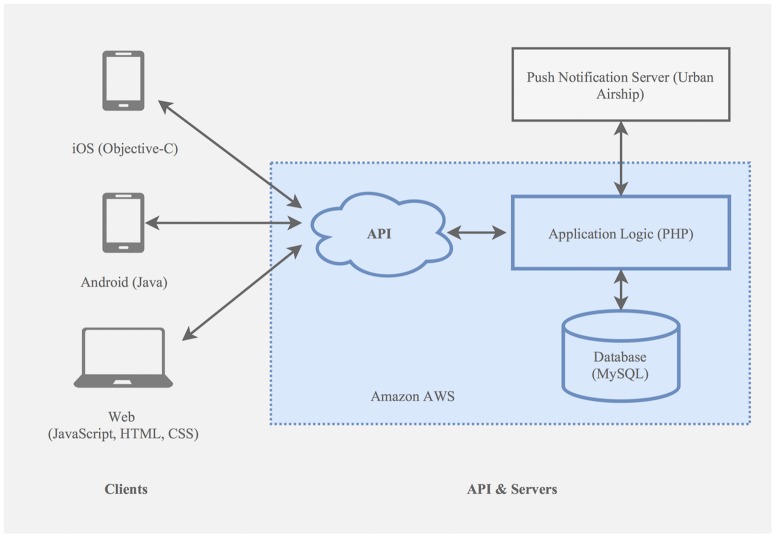
Architecture diagram.

The mobile applications were developed for iOS and Android using Java and Objective-C, respectively (Fig 1)

The web interface was based on RFC 5545 iCalendar specification. The RFC 5545 algorithm uses text-based representational sentences to keep and store information associated with recurrence rules. The algorithm assisted the administrative officer to build schedules based on rules and exceptions. This allowed the automation of a previously complex and tedious task. The software was also able to generate "alerts" about common mistakes during the scheduling process such as double booking, shift assignment during vacation, and mismatch between the department guidelines and a physician's shift schedule (S1 File).

The first prototype was tested eight months prior to the study onset with a cohort of ten medical residents who provided feedback and suggested improvements in user interface and software functionality. The mobile application was then made available on both the Apple Store (https://itunes.apple.com/us/app/escala/id998551095?) and Google Play (https://play.google.com/store/apps/details?id=br.einstein.escala).

On the date of implementation, the schedule was created by administrators with assistance of the system through the new web interface and released electronically through the mobile application to physicians. Physicians were then able to request, accept, or refuse shift exchanges among themselves without involving the administrators. As soon as an exchange was completed the schedule was updated in real time for all participants and the administrative staff and stored in the relational database.

### Questionnaire and data collection

Three electronic surveys were administered (S2 File): a baseline survey three months prior to the mobile application release, a second survey three months after implementation and third after six months of use. The surveys were designed based on questionnaires from prior studies of smartphone usage by physicians [9–12].

The data collected included demographic variables, such as age, gender, smartphone ownership, time since graduation from medical school and time working as a critical care staff physician. User experience with application installation, feedback on the mobile interface, and technical support during the study was also collected. Lastly, information regarding usage and satisfaction with the previous and the new scheduling models were obtained.

Physician satisfaction was assessed using the 4-point Likert type scale (1 = "Very unsatisfied", 2 = "Unsatisfied", 3 = "Satisfied", 4 = "Very satisfied").

The electronic surveys were done using the Google Forms online software (www.google.com/forms) sent to the participants. Completion rate was assessed weekly. For the physicians who do not complete the survey, a reminder was sent every week for 4 weeks.

### Study outcomes

The primary outcome of this study was user satisfaction of intensive care physicians at three different time points during the study: pre-implementation, and at three and six months post implementation. The secondary outcomes included time spent for and difficulty with a successful shift exchange, physician confidence with the scheduling, and user interface assessment of the new model.

### Statistical analysis

Descriptive statistics was performed as follows. Normally distributed continuous variables were summarized as mean ± standard deviation while those with a non-normal distribution were summarized as median and interquartile range. Frequencies and proportions were used for discrete variables. Only completed surveys were included in the analysis. Wilcoxon signed-rank test was employed to compare variables at two time points (survey 1 vs. survey 2; survey 1 vs. survey 3; survey 2 vs. survey 3) and the p-value threshold for level of significance was adjusted according to Bonferroni correction for multiple comparisons (significance level adjusted to 0.0167). Friedman test was used to compare degree of satisfaction in three time points. McNemar test for related frequencies was performed to compare confidence in the schedule before and after system implementation. Statistical significance level was achieved when p <0.05. Data was analyzed using IBM SPSS Statistics version 22.0 (STATACorp, Texas, USA).

## Results

A total of 51 (77%) physicians completed the baseline survey, 50 (98%) of them owned a smartphone, 32 (63%) were male, with a mean age of 37.8 ± 5.5 years (ranging from 29 to 57 years). The average time since medical school graduation was 13.6 ± 5.6 years with 10.1 ± 6.0 years dedicated to the practice of critical care specialty. Thirty-six (70%) physicians were using several routes for shift exchanges. Fifty-one (100%) of the responders were using WhatsApp, 30 (59%) regular phone call, 21 (41%) regular SMS and 8 (16%) email. Regarding the usage of the current shift exchange method(s), before the digital scheduling system implementation, close to 23 (45%) reported that they used it at least once per week, 12 (24%) fortnightly and 16 (31%) monthly.

Thirty-eight (74%) responded that it was mandatory to report all successful negotiation to the administrative office.

Prior to implementation of the scheduling system, only 20 physicians (40%) were using the mandated official department report sheet to document their shift exchange. 38 (74%) kept record in their personal notes, 22 (43%) in WhatsApp, 3 (6%) in their email; 25 (49%) were using more than one method to keep track. When asked during the baseline survey about a mobile application to perform shift exchange, the majority of responders 49 (96%) were in favor. Only 24 (47%) physicians had full confidence with how they arranged shift exchange prior to the digital scheduling system (Table 1).

**Table 1:** Surveyors baseline characteristics.

Characteristic		n = 51 (100%)
Age (years), mean (±SD)		37.8 ± 5.5
Sex (male), n (%)		32 (63)
Time since graduation (years), mean (±SD)		13.6 ± 5.6
ICU clinical practice (years), mean (±SD)		10.1 ± 6.0
Smartphone ownership (yes), n (%)		50 (98)
What method(s) are you currently using to carry out your shift exchange attempt(s)?, n (%)^a^		
	WhatsApp	51 (100)
	phone call	30 (59)
	regular SMS	21 (41)
	e-mail	8 (16)
	more than one of the above options	36 (70)
Do you need to report all successful shift exchange to the administrative staff? (Yes), n (%)		38 (74)
How often do you use the current method(s) for shift exchange?, n (%)		
	daily	6 (12)
	weekly	17 (33)
	fortnightly	12 (24)
	monthly	16 (31)
How do you record your successful shift exchange?, n (%)^a^		
	personal note	38 (74)
	WhatsApp	22 (43)
	official department sheet	20 (40)
	e-mail	3 (6)
	more than one of the above options	25 (49)
Are you interested in mobile app to manage your shift exchange attempts? (yes), n (%)		49 (96)
Do you trust in the current shift exchange model? (yes), n (%)		24 (47)

SD: standard deviation;

^a^ in this question more than one answer was possible.

49 (74%) physicians completed the second survey 3 months post-implementation. The majority 48 (98%) replied that the application installation was easy 17 (35%) or very easy 31 (63%). 46 (94%) were happy with the mobile application interface. About one third 15 (31%) of the responders did not avail of technical support. Of those who obtained technical support, the majority 27 (55%) found it to be very helpful. 90% of users reported that the application did not malfunction or only very occasionally; there was no feedback as regards unreliable functionality. When asked about the possibility of returning to the previous shift exchange method, the vast majority 47 (96%) objected. Finally, a total of 44 (90%) of responders reported they would probably 6 (12%) or certainly 38 (78%) recommend the mobile application to their colleagues (Table 2).

**Table 2:** Second survey results after 03 months of the mobile application implementation.

		n = 49 (100%)
App installation experience, n (%)		
	very easy	31 (63)
	easy	17 (35)
	not easy at all	1 (2)
App interface, n (%)		
	extremely friendly	16 (32)
	very friendly	15 (31)
	friendly	15 (31)
	very unfriendly	3 (6)
	extremely unfriendly	0 (0)
App malfunctioning, n (%)		
	constantly	3 (6)
	frequently	2 (4)
	occasionally	29 (59)
	never	15 (31)
Technical support evaluation, n (%)		
	very useful	27 (55)
	sometimes useful	4 (8)
	not useful at all	3 (6)
	did not use	15 (31)
Would you recommend the mobile application?, n (%)		
	certainly yes (100%)	38 (78)
	probably yes (75%)	6 (12)
	maybe (50%)	5 (10)
	probably not (25%)	0 (0)
	certainly not (0%)	0 (0)
Would you like to return to the previous shift exchange method? (no), n (%)		47 (96)

The third survey took place 6 months after the mobile application implementation and 9 months after the first survey. Fifty-one (77%) physicians responded, however only physicians who replied to all three surveys (regarding the degree of satisfaction) and those who answered the first and the last survey (regarding the comparison between methods) were included in the analysis. This resulted in 33 and 36 responders respectively.

33% of responders were somewhat unsatisfied and 4% very unsatisfied with the previous method for arranging shift exchange. After three months of the mobile application implementation, dissatisfaction was reduced from 33% to 4%. 35% of physicians reported they were satisfied, and 61% were very satisfied at 3 months (p<0.001).

**Fig 2:**
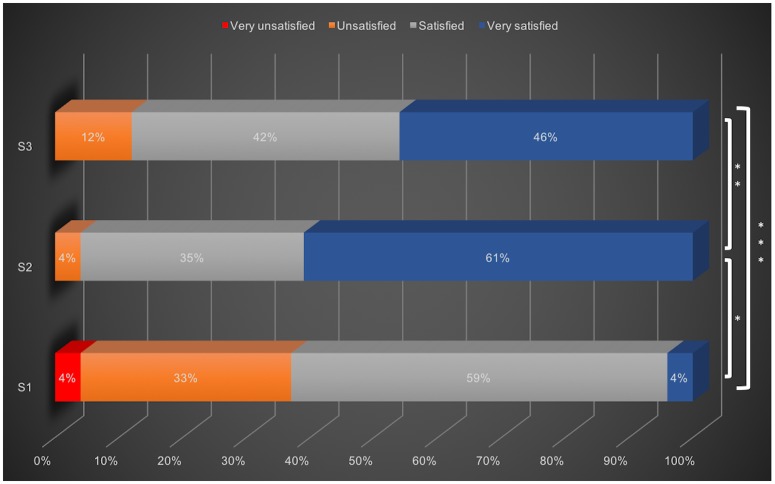
Four-point Likert satisfaction scale. S1: survey done 3 months before the mobile application implementation, S2: survey 3 months after the implementation, S3: survey done 6 months after the implementation, *p<0.001, **p = 0.52, *** p<0.001

This degree of satisfaction persisted at six months of the study, with forty-six percent reporting they were very satisfied, 42% satisfied and 12% unsatisfied. When comparing all three time points (Fig 2), the mobile application significantly increased the satisfaction of critical care physicians (p <0.001).

The mobile application also resulted in a significant reduction in the time spent for a successful shift exchange (p = 0.033). At baseline, most physicians spent hours to days (86%) to arrange a shift exchange. With the new system, 80% reported that they were able to markedly reduce time spent to just minutes or hours.

The perceived difficulty with exacting a shift swap markedly improved with the new system. In the previous method most of the responders classified that it was difficult (56%) or very difficult (6%) to arrange a successful shift exchange; with the new system 56% said that was easy and 6% very easy to perform it.

Not surprisingly there was a statistically significant improvement in the physician's trust with schedule revisions, 86% with the digital platform vs. 47% with the prior methods (p = 0.001) (Table 3).

**Table 3:** Comparison between the working shift schedule models.

		Previous model	Mobile app	p
Time spent to perform a successful shift exchange, n (%)				0.033*
	minutes	4 (11)	8 (22)	
	hours	18 (50)	21 (58)	
	days	13 (36)	6 (17)	
	weeks	1 (3)	1 (3)	
Perceived difficulty to perform a successful shift exchange, n (%)				0.049*
	very easy	0 (0)	2 (6)	
	easy	14 (39)	20 (55)	
	difficult	20 (55)	12 (33)	
	very difficult	2 (6)	2 (6)	
Do you trust in current method of shift exchange? (yes), n (%)		17 (47)	31 (86)	0.001**

*Wilcoxon signed-rank test;

**McNemar test for related proportions

## Discussion

Our study showed that a mobile application with a web interface improved and sustained physician satisfaction with shift exchanges that were done faster, more easily and more reliably. Factors that contributed to the success of the project include dissatisfaction with the previous system, the ubiquity and physician familiarity with smartphone technology, and the involvement of clinicians throughout the software development process.

The problem that was addressed by the system is one of the most under-appreciated issues in healthcare operations. Previously, the administrative staff overseeing the physician schedule faced many challenges, balancing the restrictions from work hour legislation, the department guidelines, vacation requests and at times unanticipated leaves. In addition, physicians are increasingly working at multiple sites, dealing with multiple separate schedules without any system in place to help them manage their shifts. Our study confirmed that the prior system was perceived to be inadequate.

The second factor that contributed to the success of the project was the incorporation of the mobile application. Physicians are comfortable using smartphones in many aspects of their private and professional lives [9–15]. Our cohort reported 98% ownership of smartphones; with the majority already employing “WhatsApp” to arrange shift swaps.

But the most important contributor to a successful physician adoption is the fact that physicians were involved in all stages of product development, from problem conception through beta testing. The development consisted of physicians, engineers, software developers and designers. This resulted in iterative discussions and rapid feedback loops between the users (clinicians) and the technical staff. Many have posited that one of the reasons why so few of the more than 260,000 existing health apps have led to sustained benefits is the gap between the health IT developers and clinicians [16]. Without ongoing involvement of clinicians throughout the design process, many of the apps were created based on assumptions around clinical utility and limited understanding of clinician workflow. We believe that the physician satisfaction demonstrated in this study is a testament to the value of multi-disciplinary design thinking [16,17].

### Limitations

This study has several limitations. First, it was a single center survey conducted with a relatively small number of physicians from one department. This was due to the challenges of completely overhauling the existing scheduling system in a larger group. Additionally, critical care physicians are primarily shift workers who work across multiple sites, and so innovation regarding shift exchange and management is particularly needed in this specialty.

Despite the relatively small sample size our study had a higher return rate than previously described in the literature with 77%, 73% and 73% response rates respectively over the 9-month study period [9,10].

Another potential limitation is that we didn't evaluated the administrative staff satisfaction with the web scheduling system. The development of an easy to use smart scheduling system was an important element of this project, but as the targets of our surveys were all clinician end users, we did not capture the response of administrators to the new implementation. Nonetheless, even without this specific evaluation, the impressive adoption of our solution by administrators in different departments and hospitals in the following months makes us believe that they too saw the system as an improvement over the status quo. Since the pilot study, the mobile application had more than 1400 downloads and is being used by more than 750 physicians in different specialties, across three hospitals in São Paulo, Brazil This rapid growth is likely driven by the more than 78% of the physicians who answered that they would certainly recommend our solution to their co-workers.

## Conclusion

Creating a shift schedule that accommodates the departmental requirements, labor work hour regulations and physician preferences and requests has been challenging. In this study we demonstrate that a multi-platform digital system designed by a cross-disciplinary development team significantly improved the physician experience with the complex task of scheduling. The success of this project is a testament to the value of collaborative innovation between clinicians and IT experts.

## Supporting information

S1 File The web based scheduling system. Software description.
(PDF)

S2 File Electronics surveys. (PDF)
