# Supplementary material for: Physician satisfaction with a multi-platform digital scheduling system
Source: PLoS One. 2017 Mar 22;12(3):e0174127. doi: 10.1371/journal.pone.0174127 (PMC5362101; doi:10.1371/journal.pone.0174127)

The web based scheduling software

The web scheduling software was based on RFC 5545 iCalendar specification. This specification defines an event and any repeating pattern for recurring events on calendars. It is the same tool used by well-known software calendars such as Google Calendar and Apple iCal but applied to the physician context.

For this new context we included the physician name, time period and department identification as part of the algorithm, to better identify who, when and where the physician responsible for the shift would be scheduled.

The RFC 5545 algorithm uses text-based sentences to represent recurrence rules. As an example, a sentence for a recurrence rule starting 06/13/2016, with a weekly repetition until 31/12/2016 would be:

FREQ=WEEKLY; INTERVAL=1; DTSTART=20160613; UNTIL=20161231

There are four parameters FREQ (frequency), INTERVAL (repetition interval), DTSTART (starting at) and UNTIL (ending at).

The combination of the four parameters defines the physician's allocation in the scheduling software. There are two different ways to create this allocation. The first one is a simple allocation with a start and end date on the same day. The second is recurrent allocation, which starts in a specific day and ends using one of the three following conditions: specific interval, specific date or lasting indefinitely.

The scheduling software also includes a user-friendly way to create an exception rule during the recurrent allocation allowing the administrative staff to remove specific weeks. The purpose of this last feature is to automate the process that was previously completed manually.

Apart of the allocation and exclusion features, the software was also able to generate alerts about the most common mistakes (named as "conflict" in our context) during the schedule construction, such as physician's double booking, physician assignment during vacation or leave, physician's absent booking and mismatch between the department guidelines and the physician's shift schedule (eg: same physician scheduled in two different departments at the same time or physician scheduled for a 24h shift - forbidden in our department) (Fig1)

Figure 1: The web based scheduling software diagram

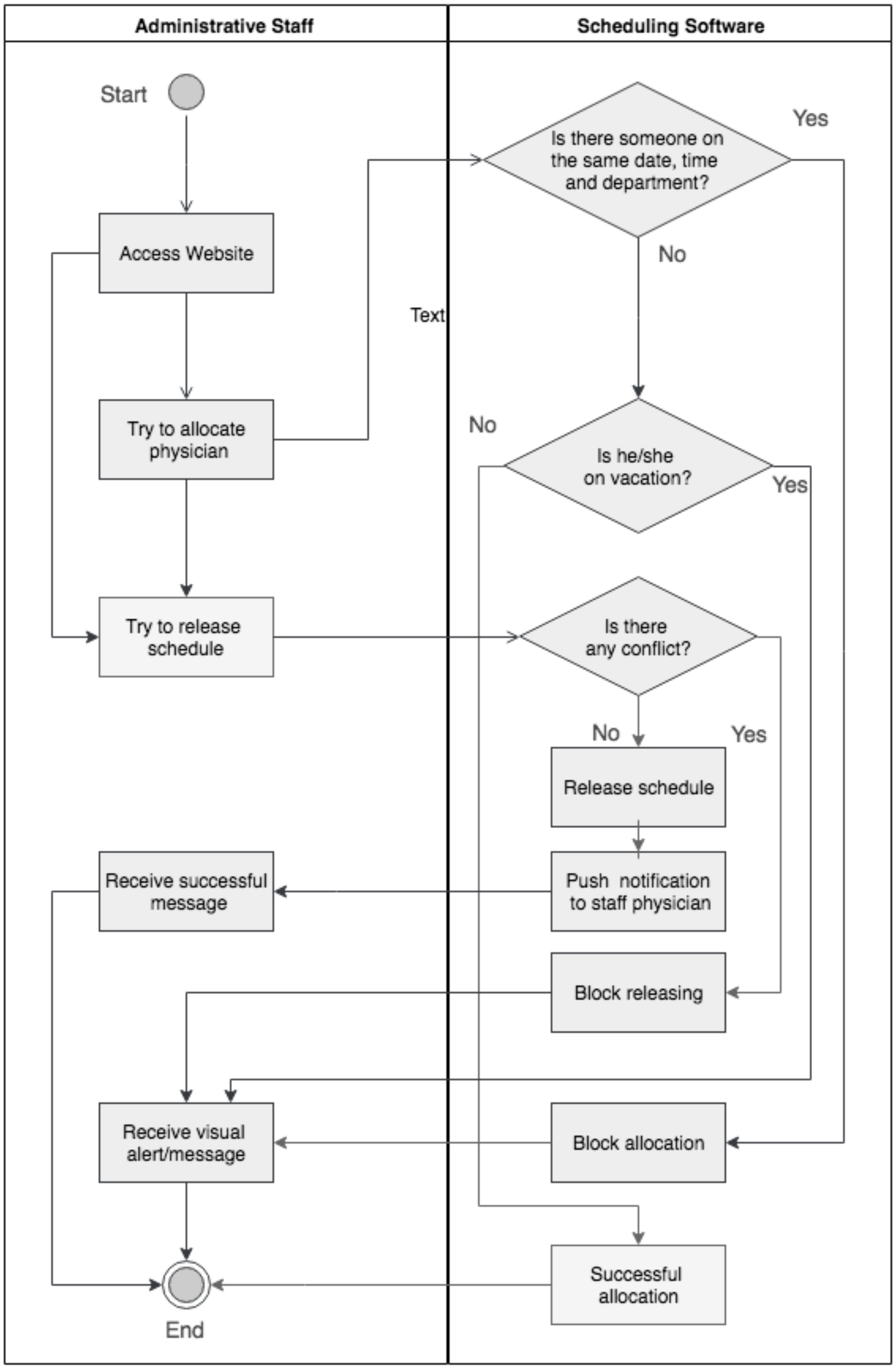

Supplement: S1 File — Software description. (PDF) [file pone.0174127.s001.pdf]
