# Supplementary material for: Physician satisfaction with a multi-platform digital scheduling system
Source: PLoS One. 2017 Mar 22;12(3):e0174127. doi: 10.1371/journal.pone.0174127 (PMC5362101; doi:10.1371/journal.pone.0174127)
Supplement: S2 File — (PDF) [file pone.0174127.s002.pdf]

Satisfaction surveys

SURVEY I/III - 3 months before the mobile application

1) What is your name?
____

2) What is your gender?
( ) Male
( ) Female

3) How old are you? (years)
____

4) How long ago did you graduate medical school? (years)
____

5) How long have you been practicing in the intensive care field?
____

6) Do you own a smartphone?
( ) Yes
( ) No

7) What method(s) are you currently using to carry out your shift exchange attempt(s) ?
* You may choose more than 1 option
( ) Whatsapp
( ) Phone call
( ) SMS
( ) Email

8) How often do you use the current method(s) for shift exchange?
( ) Daily
( ) Weekly
( ) Fortnightly
( ) Monthly

9) How do you record your successful shift exchanges?
* You may choose more than 1 option

( ) Personal note
( ) Whatsapp
( ) Official department sheet
( ) Email

10) Do you need to report all successful shift exchange to the administrative staff?
( ) Yes
( ) No

11) How much time do you usually spend to perform a successful shift exchange?
( ) Minutes
( ) Hours
( ) Days
( ) Weeks

12) What is the degree of difficulty to perform a successful shift exchange ?
( ) Very easy
( ) Easy
( ) Difficult
( ) Very difficult

13) What is your degree of satisfaction with the current shift exchange model?
( ) Very satisfied
( ) Satisfied
( ) Unsatisfied
( ) Very unsatisfied

14) Do you trust in the current shift exchange model?
( ) Yes
( ) No

15) Are you interested in a mobile app to manage your shift exchanges?
( ) Yes
( ) No
==

SURVEY II/III - 3 months after the mobile application

1) What is your name?

____

2) How was the experience installing the mobile application on your smartphone?
( ) Very easy
( ) Moderately easy
( ) It was not easy at all

3) What is your opinion about the mobile application interface?
( ) Extremely friendly
( ) Very friendly
( ) Friendly
( ) Very unfriendly
( ) Extremely unfriendly

4) How often have you experienced technical issues with the mobile application?
( ) Constantly
( ) Frequently
( ) Occasionally
( ) Never
Which one? _________________________

5) How would you rate the technical support?
( ) Very useful
( ) Sometimes useful
( ) Not very useful
( ) I did not use it

6) What is your degree of satisfaction with the current shift exchange model?
( ) Very satisfied
( ) Satisfied
( ) Unsatisfied
( ) Very unsatisfied

7) Would you recommend this mobile application to your colleagues?
( ) Very probably - 100%
( ) Most probably - 75%
( ) Maybe yes, maybe no - 50%
( ) Probably not - 25%

( ) I would not recommend it to anyone 0%

8) Would you like to return to the previous shift exchange method?
( ) Yes
( ) No
==

SURVEY III/III - 6 months after the mobile application

1) What is your name?

____

2) How much time do you usually spend to perform a successful shift exchange using the mobile application model?
( ) Minutes
( ) Hours
( ) Days
( ) Weeks

3) What is the degree of difficulty to perform a successful shift exchange using the mobile application model?
( ) Very easy
( ) Easy
( ) Difficult
( ) Very difficult

4) What is your degree of satisfaction with the current shift exchange model?
( ) Very satisfied
( ) Satisfied
( ) Unsatisfied
( ) Very unsatisfied

5) Do you trust in the current shift exchange model?
( ) Yes
( ) No

===
